# p17 Variant Expression and Evolution in HIV-Mediated Lymphomagenesis

**DOI:** 10.3390/v17040463

**Published:** 2025-03-24

**Authors:** Nicoleta Arnaut, Mark Slevin, Claudia Bănescu, Mihaela Straistă, Arnaldo Caruso, Francesca Caccuri

**Affiliations:** 1 Centre for Advanced Medical and Pharmaceutical Research, “George Emil Palade” University of Medicine, Pharmacy, Science and Technology, 540142 Targu Mures, Romania; arnaut.nicoleta99@gmail.com (N.A.); mark.slevin@umfst.ro (M.S.); claudia.banescu@umftgm.ro (C.B.); strastimira@gmail.com (M.S.); francesca.caccuri@unibs.it (F.C.); 2 Genetics Department, George Emil Palade University of Medicine, Pharmacy, Science, and Technology of Targu Mures, 540142 Targu Mures, Romania; 3 Section of Microbiology, Department of Molecular and Translational Medicine, University of Brescia, 25123 Brescia, Italy

**Keywords:** protein p17 variants, B-cell clonogenic activity, HIV-related lymphoma, next-generation sequencing

## Abstract

Non-Hodgkin lymphoma (NHL) remains the most common malignancy and cause of death among human immunodeficiency virus (HIV-1)-positive individuals, its prevalence remaining even after the introduction of combined antiretroviral therapy (cART). The mechanisms underlying B-cell tumorigenesis are still poorly understood; however, recently, a key role for p17 variants (vp17s) in lymphoma development has been clearly elucidated. Here, we describe findings on lymphomagenic vp17s and discuss their potential role as diagnostic and prognostic markers that could be used to predict the HIV-positive patients at higher risk of developing lymphoma. Specifically, vp17s endowed with amino acid (aa) insertions in their C-terminal region, at positions 114–115 (Glu-Lys), 117–118 (Ala–Ala) and 125–126 (Gly–Asp), were found to be significantly more prevalent in HIV-positive individuals with lymphoma as compared to those without. Alterations in the primary aa sequences destabilize the protein, exposing a previously hidden functional epitope which interacts with protease-activated receptor-1 (PAR-1) and stimulates the protein kinase B pathway, conferring oncogenic potential to vp17s and possibly contributing to lymphomagenesis. Therefore, ultradeep sequencing technologies, such as next-generation sequencing, could serve as a valuable screening tool for identifying and monitoring the HIV-positive patients at higher risk of developing lymphoma, paving the way for targeted preventive intervention strategies.

## 1. Introduction

The early initiation and effective application of combined antiretroviral therapy (cART) has significantly improved the life expectancy of individuals with human immunodeficiency virus type 1 (HIV-1), leading to a near-normal lifespan [1]. As a result, this prolonged longevity has led to an increasing incidence of several comorbidities, the most common being hepatitis, mental disorders and cardiovascular diseases [2]. On the other hand, cART has reduced the incidence of acquired immunodeficiency syndrome (AIDS)—defining cancers, making them less common co-occurring conditions. However, even after the introduction of cART, individuals living with HIV are 11.5 times more likely to develop non-Hodgkin lymphoma (NHL) compared to the general population. Currently, NHL is the most frequent cancer-related comorbidity in HIV and the leading cause of death among HIV-1-positive patients [3,4].

The mechanisms underlying B-cell tumorigenesis remain poorly understood. Previously, an indirect role of HIV, via induced immunosuppression, co-infection with oncoviruses, chronic antigenic stimulation and cytokine dysregulation, has been proposed. However, these processes do not fully explain the elevated risk of developing lymphomas in cART-responsive patients who have attained normal immune reconstitution [5]. More recent evidence supports a direct contribution of HIV to lymphomagenesis, independently of immunodeficiency, through specific virus products/proteins that are released systemically and accumulate in lymphoid tissues, even in aviremic patients [6].

Particular attention has been directed toward the HIV-1 matrix protein p17 (p17), which can undergo mutations, leading to the generation of different variants (vp17s). Research has shown that specific vp17s are significantly more prevalent in HIV-positive individuals with lymphoma compared to those without. These vp17s display clonogenic activity in B-cells, suggesting that specific molecular signatures may confer this potential [7].

Here, we describe the role of vp17s in lymphomagenesis and hypothesize the molecular signatures associated with their clonogenic activity in B-cells. Furthermore, we evaluate their potential utility in identifying patients at risk of HIV-related lymphoma, providing insights into possible diagnostic, prognostic and preventive strategies.

## 2. Characterization, Production and Tissue Accumulation of p17

p17 is a 132-amino acid (aa) structural protein, encoded by the GAG gene, that plays a critical role in most stages of the HIV-1 life cycle. The protein is partially globular, being composed of four helixes that form a dense central domain, topped by a β-sheet containing basic residues. These residues, which are highly conserved across various HIV-1 strains, play a key role in cell membrane binding. The fifth helix at the C-terminal region extends outward, exposing functional elements critical for the early stages of HIV-1 infection. This structural arrangement is stabilized by secondary interactions, primarily through hydrogen bonds [8].

It was originally believed that p17 is produced exclusively during active viral replication, through the protease cleavage of the GAG precursor polyprotein [9]. However, more recent studies have indicated that HIV-1 GAG gene transcription can be triggered by various stimuli, including histone deacetylase inhibitors or prostratin, a nuclear factor -κB inducer [10], even in the presence of protease inhibitors [11]. Furthermore, the synthesized GAG precursor polyprotein, upon binding to phosphatidylinositol-(4,5)-bisphosphate (PIP2), undergoes cleavage by aspartyl proteases. Through this unconventional pathway, biologically active p17 is secreted into the extracellular space, even in the absence of ongoing virus replication [12,13].

Virion-free p17 has been detected in the peripheral blood of individuals successfully treated with cART, at nanomolar concentrations [14]. Notably, plasma p17 concentrations are heterogeneous and entirely independent of a patient’s viremic status [15]. p17 can cross the blood–brain barrier (BBB), potentially forming toxic amyloidogenic assemblies within the central nervous system and contributing to HIV-1-associated neurocognitive disorders [16,17]. Moreover, p17 has been identified in the germinal centers of lymph nodules in cART-treated patients with undetectable virus replication [18]. One possible explanation is that latently HIV-infected resting CD4+ cells could produce p17 when stimulated with interleukin (IL)-7 or CD3/28 [19].

These findings support the theory that p17 can be produced and released from cells in the absence of active viral replication. Consequently, it may chronically persist and accumulate in the lymph node microenvironment, potentially contributing to lymphoma’s development, even in individuals with a pharmacological suppression of viral replication [8].

## 3. Impact of C- and N-Terminal Mutations on p17 Stability and Activity

### 3.1. Potential Factors Driving Generation of p17 Mutations

HIV-1 constantly mutates due to its high replication rate, the error-prone nature of RNA polymerase, susceptibility to natural selection, as well as drug and immunological pressures. All these factors influence its genetic diversity, allowing the virus to adapt to the host environment and leading to persistent changes in protein structure and function [20,21]. Although p17 has long been regarded as having a highly preserved and stable aa structure, recent studies have revealed a greater sequence diversity within a same patient than was previously expected. When HIV-1 subtype B-RNA from plasma samples of 18 patients was analyzed using ultradeep pyrosequencing, multiple variants of p17 were found at different frequencies in nearly all patients. Whilst the mutations were distributed throughout the entire viral protein, they were more commonly detected in the C-terminal region [22].

These findings suggest that p17 derived from clade B virus (BH10), referred to as reference p17, can also undergo mutations during the natural course of infection, particularly in the C-terminal region. This results in various vP17s, which may help the virus to evade immune detection, since p17 is an important target for both antibody- and cell-mediated immune responses. Furthermore, the pressure exerted by the host immune system could drive the selection and expansion of different vp17s. Additionally, conformational changes in p17 might enhance the virus’s resistance to protease inhibitors [7].

A recent study revealed that mutations in the reference p17 can occur due to the inefficiency of HIV-1 reverse transcriptase in processing specific regions of the GAG gene, especially those containing inverted repeats and palindromic sequences at the C-terminal region of the matrix protein, which causes the enzyme to pause during replication. This stalling can lead to errors, because reverse transcriptase may resume replication from an incorrect position, resulting in insertions or deletions. Concomitant recombination events might contribute to the formation of vp17s with altered biological functions, potentially influencing lymphoma development in individuals living with HIV-1 [23].

### 3.2. The Impact of C-Mutations

Although it may seem intuitive to hypothesize that the mutated C-terminal region directly interacts with receptors to exert biological activity, a more plausible explanation is that alterations in the primary aa sequence reorganize the secondary structure, leading to significant conformational shifts, destabilization, misfolding and even the acquisition of new functions. By nuclear magnetic resonance (NMR) spectroscopy and X-ray analysis Giagulli et al. showed that the C-terminal region of p17 does not directly bind to the cellular receptor, but, instead, that it was in close proximity to the globular head, which contains the functional epitope AT20 (aa 9 to 28)—a well-known loop critical for the biological activity of the reference p17. In this way, the C-terminal region, particularly helix H5, with its high flexibility, modulates the interaction between the globular head and the receptors through steric hindrance mechanisms [8].

Notably, in reference p17-treated Raji cells (a human B-cell line expressing the p17 receptor on their membrane), the protein kinase B (Akt) pathway, which is responsible for B-cell growth and clonogenicity, was found to be inhibited [8]. In contrast, the C-terminal truncated form, p17Δ36, significantly activated the Akt pathway, leading to B-cell clonogenic activity. This observation led to the hypothesis that an N-terminal epitope of p17 may be responsible for activating the Akt pathway, but in the adequately folded state it is masked by the C-terminal region through steric hindrance. Therefore, in vp17s a conformational shift exposes this epitope, triggering subsequent clonogenic activity in B-cells [8].

A further study compared the reference p17 with a specific highly biologically active variant S75X, originating from a Ugandan HIV-1 strain (subtype A1), which carries a single Arginine to Glycine mutation at position 76. Molecular dynamics simulations, combined with circular dichroism (CD) and NMR spectroscopy, revealed that this mutation disrupted the hydrogen bond network, leading to conformational changes that exposed a previously hidden functional epitope in the N-terminal region of reference p17, although its exact position remained unknown. The exposed epitope subsequently interacted with an unrecognized receptor, promoting B-cell clonogenic activity, as confirmed by in vitro clonogenic assays [24].

### 3.3. Identification of Clonogenic Epitope of vp17 and Its RECEPTOR

The epitope’s precise localization was elucidated when He et al. induced a disulfide bond between the Cys residues at positions 57 and 87 in the reference p17, generating a destabilized variant capable of stimulating B-cell proliferation. Further protein dissection identified the functional epitope, corresponding to aa 1–20 in the N-terminal region, which overlaps with the AT20 domain. A synthetic 20 aa peptide, F1, imitating the active epitope, demonstrated the ability to promote B-cell growth and proliferation in vitro [25].

In proof-of-concept experiments, clonogenic vp17s derived from HIV-positive NHL patients, containing aa insertions at positions 117–118 and 125–126, had three positively charged aas—Arg15, Lys18 and Arg20—replaced with negatively charged Asp. Subsequent in vitro studies showed that these engineered vp17s, unlike their natural counterparts, no longer exhibited a clonogenic activity on B-cells. These findings imply that such critical residues are more exposed in vp17s compared to the reference p17, which may contribute to their newly acquired clonogenic effects on B-cells [25].

Additionally, the synthetic F1 peptide did not interact with the recognized receptors of the reference p17, chemokine (C-X-C motif) receptor (CXCR)-1 and CXCR-2, indicating that a different receptor might mediate the B-cell growth-enhancing effects of vp17s [25]. Subsequent studies identified this receptor as protease-activated receptor 1 (PAR1) [26], which is part of the extensive family of G protein-coupled receptors and is widely expressed on various cell types, including immune and epithelial cells, vascular myocytes and macrophages within the tumor microenvironment. Its activation contributes to the stimulation of the Akt pathway, which subsequently regulates the expression of several molecules, including RAC1, ABL1, p53 and CDK1, leading to enhanced cell proliferation, tumor progression and metastatic dissemination [26,27].

### 3.4. The Impact of N-Terminal Region Mutations

Mutations in the C-terminal region are not the sole contributors to p17 destabilization. Research has shown that the clonogenic functional epitope within the N-terminal region is surrounded by hydrophobic aas, and its structural integrity relies on the hydrophobic cluster (HC)-AT20, which is maintained by Trp16 and Tyr29. A single aa mutation within the N-terminal globular domain can also trigger conformational changes, leading to partial misfolding, the destabilization of HC-AT20 and increased exposure of the clonogenic functional epitope. This makes the epitope more accessible for binding to PAR-1, thereby promoting B-cell growth and proliferation. Computational alanine scanning confirmed the pivotal role of Trp16 and Tyr29 in maintaining the structural integrity and function of p17 [28].

The reference p17 was modified by substituting Trp16 and Tyr29, either separately or together, with Ala, and a single-cell clonogenic assay was performed using Raji cells. The mutated p17s significantly enhanced B-cell proliferation compared to cells treated with reference p17 or nontreated cells, with the effect being similar to that observed in cells treated with vp17s carrying aa insertions at positions 117–118 [28].

**Figure 1:**
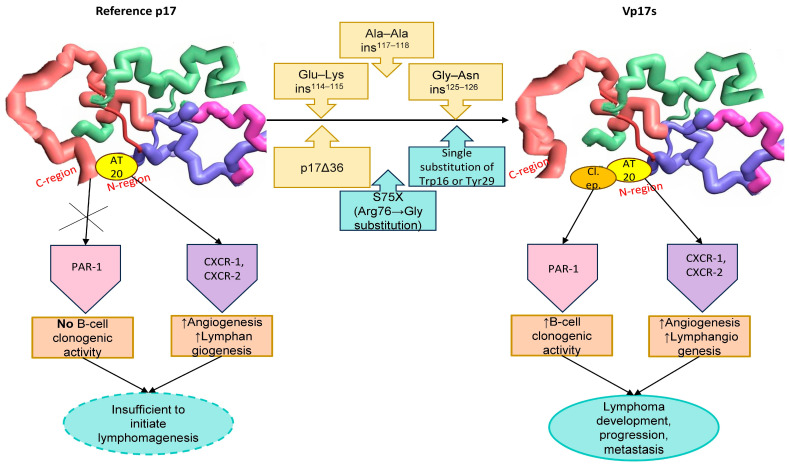
Impact of amino acids alterations on protein conformation and activity. In its adequately folded state, reference p17 shows no biological activity regarding B-cell growth, as the clonogenic epitope (Cl. ep.) is masked by the C-terminal region through steric hindrance. It exhibits only proangiogenic and lymphangiogenic effects mediated by the AT20 epitope. In contrast, vp17s, characterized by different alterations within both the C-terminal and N-terminal regions, undergo conformational changes and become destabilized, with a subsequent exposure of the functional epitope, which exerts B-cell clonogenic activity. In combination with the pro-vasculogenic activity, this establishes a microenvironment that may trigger and sustain lymphoma initiation, progression and metastasis.

These findings suggest that potential mutations within both the C-terminal and N-terminal regions may lead to destabilization, misfolding or even complete unfolding, resulting in the subsequent exposure of the clonogenic functional epitope located within the globular head (aa 1–20). This most likely provides the molecular basis for the contrasting properties of reference p17 (which shows no biological activity regarding B-cell growth), and vp17s, which exhibit significant B-cell clonogenic activity (Figure 1).

## 4. p17 Mutations in Lymphoma Patients

Dolcetti et al. analyzed the p17 gene sequence in tumor tissue and plasma samples from ten HIV-positive patients: five with NHL and five without. Ultradeep pyrosequencing revealed that the intrasample diversity of vp17s was markedly greater in patients with lymphoma compared to those without it. In particular, vp17s with C-terminal insertions at positions 117–118 (Ala–Ala) or 125–126 (Gly–Asn or Gly–Gln–Ala–Asn–Gln–Asn) were more prevalent in the plasma of HIV+ individuals with NHL, sharing the same structure as those found in tissue samples. Circular dichroism spectroscopy and thermal denaturation showed that the Gly–Asn insertion at positions 125–126 stabilized p17, while the Ala–Ala insertion at positions 117–118 destabilized it, leading to unfolding and conformational changes [29].

A subsequent in vitro study on the ability of reference p17 and vp17s derived from lymphoma and non-lymphoma patients to stimulate clonogenic activity in Raji cells indicated that the vp17 with a 117–118 Ala–Ala insertion significantly enhanced the number of cell colonies in soft agar at concentrations ranging from 0.05 to 0.2 µg/mL, unlike reference p17 and vp17 from non-lymphoma patients. Western blot analysis of Raji lysates indicated that the underlying mechanism was consistent with those previously described [29].

Although earlier research concluded that the insertion of 125–126 Gly–Asn alone did not induce clonogenic activity, implying that other mutations or structural elements may explain its high prevalence in lymphoma patients, recent in vitro studies have shown that the vp17 with this mutation, like other clonogenic vp17s, can bind to PAR1, activating the Akt pathway and triggering molecular mechanisms distinct from those induced by the 117–118 Ala–Ala variant, thereby promoting B-cell colony growth [26].

A large retrospective study further assessed the prevalence of vp17s in plasma samples from two cohorts of HIV-1 positive patients: 83 with lymphoma and 70 without. The results confirmed a considerably higher prevalence of vp17s in lymphoma patients (49.4%) compared to those without (35.7%). Sanger sequencing showed a diverse vp17 pattern in the control group, characterized by multiple aa insertions at various positions within the C-terminal region. In contrast, lymphoma patients displayed a more defined and distinct pattern of aa insertions, consistently located within the C-terminal region, between residues 114 and 130, with the most frequent insertions occurring at positions 117–118 (23% of patients) and 125–126 (15% of patients). However, 50.6% of lymphoma patients did not exhibit a dominant vp17, indicating that a subdominant vp17 may also play a significant role in lymphoma development. The distribution of vp17s was similar across different lymphoma histotypes, suggesting that vp17s alone may not serve as markers for distinct HIV-1–associated NHL subtypes or HL [7].

In the same study, genomic surveillance identified a previously unrecognized vp17, characterized by a Glu-Lys insertion at positions 114–115, in 4% of the control cohort. Subsequently, Raji cells were cultured with or without this vp17. The colony formation assay confirmed its growth-promoting and clonogenic activities on B-cells, in contrast to the untreated or reference p17-treated cells, suggesting that this vp17 may contribute to the further development of lymphoma [7].

The additional examination of 3990 sequences belonging to the HIV-1 clade B, deposited from 1985 to 2017, revealed a global increase over time in the prevalence of HIV-1 mutants expressing vp17s with aa insertions at positions 117–118. These mutations were fixed and passed on to progeny virions, highlighting the progressive, global spread of mutant viruses expressing B-cell clonogenic vp17s, which may eventually replace the virus expressing reference p17 [7].

Based on these findings, the Ala–Ala insertion at positions 117–118 and the Gly–Asn insertion at positions 125–126, being most prevalent in HIV-positive patients with lymphoma, as well as the Glu-Lys insertion at positions 114–115 (Figure 1) should be regarded as molecular signatures of vp17s with B-cell growth-promoting and clonogenic activity. These could serve as potential predictors of lymphoma development and/or as markers to detect individuals at high risk of developing lymphoma.

## 5. vp17-Mediated Signaling Mechanisms in Lymphomagenesis

### 5.1. vp17 Pro-Angiogenic and Pro-Lymphangiogenic Effects

Studies have demonstrated that p17 exhibits IL-8-like chemokine effects, particularly pro-angiogenic and pro-lymphangiogenic, through the binding of its functional region AT20 to both IL-8 receptors: CXCR-1 and CXCR-2 [30,31].

In vitro and in vivo studies have confirmed that p17, at a nanomolar concentration (10 ng/mL), interacted with a high affinity with CXCR-1 and CXCR-2 on endothelial cells (EC), activating small G proteins and subsequently stimulating the phosphoinositide 3-kinase (PI3K)/Akt and mitogen-activated protein kinase/extracellular-signal-regulated kinase 1/2 signaling cascades. Both pathways are essential and may work synergistically to facilitate the formation of capillary-like structures. Thus, p17 strongly promoted aberrant angiogenesis, resembling the pro-vasculogenic activity of VEGF-A. This finding was further supported by the detection of virion-free p17 within the nuclei of EC in the blood vessels of HIV-1 patients [32].

**Figure 2:**
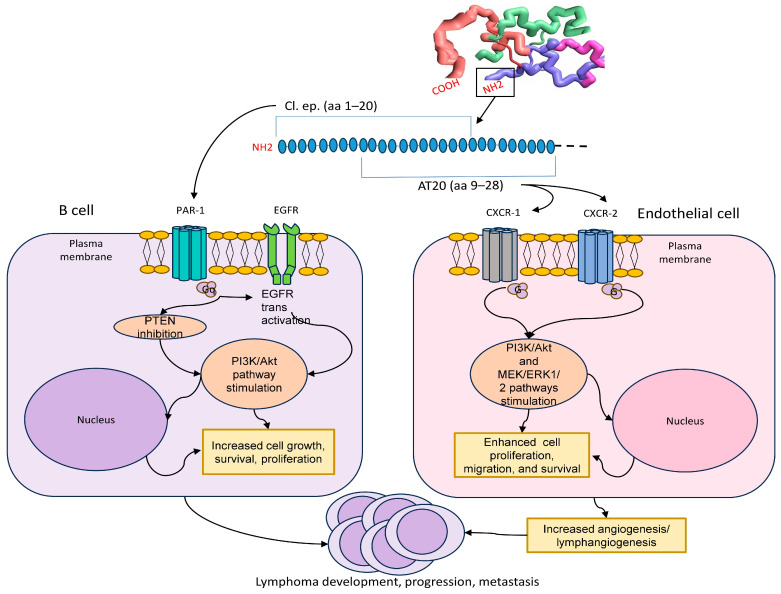
Vp17-mediated signaling mechanisms in lymphomagenesis. Vp17s may contribute to lymphomagenesis through a “dual-component pathway”, exerting pro-angiogenic and pro-lymphangiogenic effects on endothelial cells, mediated by AT20, while promoting B-cell clonogenic activity via the clonogenic functional epitope (Cl. ep.).

By interacting with CXCR-1 and CXCR-2 p17 also exerts pro-lymphangiogenic effects at nanomolar concentrations. An in vitro model using lymphatic EC, originating from the human lymph nodes, showed that the CXCR-1 and CXCR-2 stimulation of the Akt-dependent ERK pathway was enhanced and then maintained by a secondary endothelin-1/endothelin receptor B interaction. Since endothelin-1 is a key gene associated with activated lymphatic EC isolated from metastatic lymph nodes, these signaling interactions may serve to enhance and extend the lymphangiogenic activity of p17 within the (pre) tumor microenvironment. The Matrigel plug assay confirmed that p17 promoted the expansion of lymphatic vessels in vivo, providing evidence that it could directly influence lymphatic vessel formation [33] (Figure 2).

Another in vivo study compared the angiogenic and lymphangiogenic potential of reference p17 and the S75X variant. The results showed that whilst both proteins stimulated angiogenesis and lymphangiogenesis, the S75X variant induced more structured vessel formation with evidence of patency and functional capacity when compared to reference p17, as demonstrated by histological and immunofluorescence analyses [34].

These findings support the idea that mutation-induced destabilization and misfolding enhance the exposure of the globular head, facilitating a stronger interaction between AT20 and CXCR-1 and CXCR-2, which results in both increased pro-angiogenic and pro-lymphangiogenic effects. Angiogenesis and lymphangiogenesis are crucial for sustaining lymphoma proliferation and survival, along with promoting its metastasis [35,36].

### 5.2. Understanding vp17 Signaling Pathways Mediating the Clonogenic Effects in B-Cells

As previously discussed, reference p17 (genotype B strain BH10) and some vp17s from HIV+ patients without lymphoma did not exhibit, in vitro, clonogenic activity on B-cells [8,29]. By contrast, whether synthesized experimentally (such as the C-terminal 36-aa-long truncated forms of p17 [8]) or found in HIV-positive patients (e.g., the S75X variant with an Arginine-to-Glycine substitution at position 76 [24], the vp17 with a Glu-Lys insertion at positions 114–115 [7], or the vp17s from lymphoma patients with Ala–Ala insertion at positions 117–118 or Gly–Asn insertion at positions 125–126 [7,26,29,37]), all of these variants showed increased B-cell clonogenic potential in vitro, which was induced by phosphatase and tensin homolog (PTEN) inhibition, epidermal growth factor receptor (EGFR) transactivation and subsequent Akt pathway stimulation. This cascade further modulates several molecules, including RAC1, ABL1, p53 and CDK1, which play pivotal roles in regulating cell proliferation, survival, tumor progression and metastasis [26] (Figure 2).

Phosphatidylinositol 3,4,5-trisphosphate (PIP3), synthesized by PI3K, is essential for Akt activation, facilitating its translocation to the cell membrane. PTEN, a lipid phosphatase, dephosphorylates PIP3 to PIP2, thereby reducing the PIP3 levels and thus effectively suppressing the Akt pathway [38]. Conversely, EGFR activation enhances PI3K activity, resulting in increased PIP3 levels and subsequent Akt up-regulation [39].

Recent in vitro studies on Raji cells, using Western blot, phosphoproteomic and bioinformatic analyses, showed that the clonogenic activity of vp17s is driven by their interaction with PAR1, followed by Gq protein activation. This induces the Ser/Thr phosphorylation of PTEN, inhibiting its function and simultaneously triggering EGFR transactivation. Consequently, the suppression of PTEN and stimulation of EGFR lead to elevated PIP3 levels, resulting in the increased phosphorylation of Akt with its subsequent activation. This cascade has been shown to promote Raji cell proliferation, supporting its potential B-cell tumorigenic role in vivo [8,24,26,29]. Additionally, a synthetic F1 peptide (aa 2–21 of the viral protein), corresponding to the clonogenic epitope of vp17s, has been shown to effectively bind PAR1 and activate the PAR1/EGFR/PI3K/Akt pathway. This validates the concept that the exposed functional epitope plays a crucial role in driving the clonogenic activity of vp17s [26].

Taken together, the pro-angiogenic and pro-lymphangiogenic effects, mediated by the interaction between AT20 (aa 9–28) and both CXCR1 and CXCR2, along with the B-cell clonogenic activity, sustained by the binding of a the clonogenic functional epitope (aa 1–20) to PAR-1, explain the capacity of vp17s to promote lymphomagenesis through a “dual-component pathway”. In combination with the pro-vasculogenic activity, this establishes a microenvironment that may trigger and sustain lymphoma initiation, progression and metastasis. By contrast, compared to clonogenic vp17s, reference p17 exhibits lower pro-angiogenic and pro-lymphangiogenic activities and does not promote B-cell clonogenicity; therefore, its activity is insufficient to initiate lymphomagenesis.

## 6. Diagnostic Methods for Risk Detection and Prognosis

Ultradeep sequencing technologies, such as next-generation sequencing (NGS), represent a valuable approach that enables the detection of minor viral variants at frequencies as low as 1%, due to their rapid data generation and high-throughput capacity [40]. The analysis of results can be further optimized using specialized software or advanced tools, including artificial intelligence (AI). NGS can be used to monitor the evolution of vp17 quasispecies in HIV-1-positive patients, comparing those with and without lymphoma, to assess the extent to which their proportion increases over time within the same individual or patient, and potentially leading to the replacement of the reference p17. By statistically correlating variant types with clinical histories NGS might be used to detect and quantify the oncogenic potential of vp17s, linking these variants to clinical outcomes.

A well-defined NGS protocol could be periodically applied to the same HIV-positive individual to monitor the emergence of clonogenic mutations within the reference p17, serving as a longitudinal and dynamic screening platform for identifying molecular changes over time, thereby enhancing the ability to predict lymphoma development. Integrating this approach into routine molecular diagnostics could provide a robust screening tool for identifying HIV-positive patients at high risk of developing lymphoma, paving the way for targeted preventive interventions.

This screening would be of critical importance in regions where cART is not readily accessible, as patients in these areas may face compounded risks due to limited therapeutic options. Moreover, given the persistently elevated risk of developing lymphoma, even in patients successfully treated with (cART), it is imperative to extend screening efforts to this population. Implementing targeted screening programs in both settings could significantly improve early detection and intervention, ultimately reducing the morbidity and mortality associated with lymphoma in HIV-positive populations.

## 7. Future Perspectives

Future studies should examine larger cohorts of HIV-positive individuals, both with and without lymphoma, to identify dominant and particularly subdominant vp17s endowed with B-cell clonogenic activity, taking into account that half of all lymphoma patients examined to date did not exhibit a dominant vp17.

Also, an intriguing prospect for future research would be to explore whether these HIV-1 variants possess an enhanced adaptability to the human host compared to the wild-type strains and/or reflect an evolutionary response to cART [7]. A further step would be to assess and characterize in vivo the ability of oncogenic vp17s to promote lymphoma development, growth and aggressiveness/metastasis, utilizing various vectors, such as lentiviral vectors, to generate transgenic mice [41,42].

Once the clonogenic potential of vp17 is established in vitro and in vivo, subsequent studies might focus on determining their three-dimensional structure, identifying key residues involved in modulating epitope accessibility and exploring potential druggable pockets. These insights would be essential for developing preventive/therapeutic strategies, such as small molecules, to block vp17s binding to host cells and, consequently, inhibit their clonogenic activity. In this context, the integration of molecular dynamic simulations, AI and machine learning will be valuable in identifying new drugs or repurposing existing ones that could effectively target the druggable pockets [28]. An alternative approach could involve disrupting the Pr55Gag/PI(4,5)P2 interaction using small-molecule inhibitors that target and occupy the PI(4,5)P2 binding site on the viral protein [43].

In this regard, a mouse IgG anti-p17 monoclonal antibody (mAb), MBS-3, targeting a linear epitope (aa 9–18) within the AT20 functional domain completely inhibited p17 binding to Raji cells, whilst another mAb, MK-18, which binds to the p17 C-terminal domain (aa 115–132) exhibited strong neutralizing effects, likely due to steric hindrance [8]. These findings pave the way for using antibodies to block the binding of the functional AT20 epitope to its receptors, CXCR1 and CXCR2, in order to prevent their pro-angiogenic and pro-lymphangiogenic effects. Additionally, they create opportunities for developing antibodies that could inhibit the interaction with PAR-1, preventing B-cell clonogenic and oncogenic activities.

Moreover, in cART-treated patients, the AT20-based therapeutic vaccine for HIV-1 has been shown to induce and maintain a durable, neutralizing antibody-mediated response against the functional epitope AT20 (aa 9–28; responsible for pro-angiogenic and pro-lymphangiogenic effects) and has successfully completed phase I clinical trials [44,45]. A subsequent study demonstrated that these vaccine-induced anti-AT20 antibodies can neutralize the clonogenic activity associated with a vp17, in which Trp16 and Tyr29 were mutated to alanine [28].

Taking into consideration the above findings, it is worth considering whether through the administration of small molecules the passive transfer of synthesized antibodies or active vaccination, both mechanisms of the “dual-component pathway” (the interaction of AT20 with CXCR-1 and CXCR-2 and the interaction of clonogenic epitope with PAR-1), could or should be targeted to most effectively prevent vp17-induced and sustained lymphomagenesis. If successful, these approaches may represent potential as a preventive strategy to reduce or eliminate the risk of developing lymphomas in HIV+ patients.

## 8. Conclusions

Vp17s can be produced and released from cells through pathways, independent of active viral replication, accumulating and persisting within the lymph nodes of HIV-infected individuals. Alterations in the primary amino acid sequences, particularly insertions in the C-terminal region, destabilize the protein, exposing a previously hidden N-terminal clonogenic epitope, which overlaps with the AT20 domain. This epitope promotes B-cell growth and proliferation, while AT20 exhibits pro-angiogenic and pro-lymphangiogenic properties, establishing specific vp17s as potential contributors to lymphomagenesis through a “dual-component pathway”. Therefore, ultradeep sequencing technologies, such as NGS, could detect and quantify the oncogenic potential of vp17s, serving as a valuable screening tool for identifying and monitoring HIV-positive patients at high risk of developing lymphoma, and paving the way for targeted preventive interventions.

## References

[1] Trickey A., Sabin C.A., Burkholder G., Crane H., d’Arminio Monforte A., Egger M., Gill M.J., Grabar S., Guest J.L., Jarrin I. (2023). Life Expectancy after 2015 of Adults with HIV on Long-Term Antiretroviral Therapy in Europe and North America: A Collaborative Analysis of Cohort Studies. Lancet HIV.

[2] Lorenc A., Ananthavarathan P., Lorigan J., Jowata M., Brook G., Banarsee R. (2014). The Prevalence of Comorbidities among People Living with HIV in Brent: A Diverse London Borough. Lond. J. Prim. Care.

[3] Hernández-Ramírez R.U., Shiels M.S., Dubrow R., Engels E.A. (2017). Cancer Risk in HIV-Infected People in the USA from 1996 to 2012: A Population-Based, Registry-Linkage Study. Lancet HIV.

[4] Morlat P., Roussillon C., Henard S., Salmon D., Bonnet F., Cacoub P., Georget A., Aouba A., Rosenthal E., May T. (2014). Causes of Death among HIV-Infected Patients in France in 2010 (National Survey): Trends since 2000. AIDS.

[5] Pantanowitz L., Carbone A., Dolcetti R. (2015). Microenvironment and HIV-Related Lymphomagenesis. Semin. Cancer Biol..

[6] Dolcetti R., Gloghini A., Caruso A., Carbone A. (2016). A Lymphomagenic Role for HIV beyond Immune Suppression?. Blood.

[7] Caccuri F., Messali S., Zani A., Campisi G., Giovanetti M., Zanussi S., Vaccher E., Fabris S., Bugatti A., Focà E. (2022). HIV-1 Mutants Expressing B Cell Clonogenic Matrix Protein P17 Variants Are Increasing Their Prevalence Worldwide. Proc. Natl. Acad. Sci. USA.

[8] Giagulli C., Marsico S., Magiera A.K., Bruno R., Caccuri F., Barone I., Fiorentini S., Andò S., Caruso A. (2011). Opposite Effects of HIV-1 P17 Variants on PTEN Activation and Cell Growth in B Cells. PLoS ONE.

[9] Fiorentini S., Marini E., Caracciolo S., Caruso A. (2006). Functions of the HIV-1 Matrix Protein P17. New Microbiol..

[10] Reuse S., Calao M., Kabeya K., Guiguen A., Gatot J.-S., Quivy V., Vanhulle C., Lamine A., Vaira D., Demonte D. (2009). Synergistic Activation of HIV-1 Expression by Deacetylase Inhibitors and Prostratin: Implications for Treatment of Latent Infection. PLoS ONE.

[11] Vandergeeten C., Quivy V., Moutschen M., Van Lint C., Piette J., Legrand-Poels S. (2007). HIV-1 Protease Inhibitors Do Not Interfere with Provirus Transcription and Host Cell Apoptosis Induced by Combined Treatment TNF-Alpha + TSA. Biochem. Pharmacol..

[12] Caccuri F., Iaria M.L., Campilongo F., Varney K., Rossi A., Mitola S., Schiarea S., Bugatti A., Mazzuca P., Giagulli C. (2016). Cellular Aspartyl Proteases Promote the Unconventional Secretion of Biologically Active HIV-1 Matrix Protein P17. Sci. Rep..

[13] Bugatti A., Caccuri F., Filippini F., Ravelli C., Caruso A. (2021). Binding to PI(4,5)P2 Is Indispensable for Secretion of B-Cell Clonogenic HIV-1 Matrix Protein P17 Variants. J. Biol. Chem..

[14] Fiorentini S., Riboldi E., Facchetti F., Avolio M., Fabbri M., Tosti G., Becker P.D., Guzman C.A., Sozzani S., Caruso A. (2008). HIV-1 Matrix Protein P17 Induces Human Plasmacytoid Dendritic Cells to Acquire a Migratory Immature Cell Phenotype. Proc. Natl. Acad. Sci. USA.

[15] Zani A., Messali S., Uggeri M., Bonfanti C., Caruso A., Caccuri F. (2024). Detection of HIV-1 Matrix Protein P17 in Sera of Viremic and Aviremic Patients. J. Virol. Methods.

[16] Caccuri F., Neves V., Gano L., Correia J.D.G., Oliveira M.C., Mazzuca P., Caruso A., Castanho M. (2022). The HIV-1 Matrix Protein P17 Does Cross the Blood-Brain Barrier. J. Virol..

[17] Zeinolabediny Y., Caccuri F., Colombo L., Morelli F., Romeo M., Rossi A., Schiarea S., Ciaramelli C., Airoldi C., Weston R. (2017). HIV-1 Matrix Protein P17 Misfolding Forms Toxic Amyloidogenic Assemblies That Induce Neurocognitive Disorders. Sci. Rep..

[18] Popovic M., Tenner-Racz K., Pelser C., Stellbrink H.-J., van Lunzen J., Lewis G., Kalyanaraman V.S., Gallo R.C., Racz P. (2005). Persistence of HIV-1 Structural Proteins and Glycoproteins in Lymph Nodes of Patients under Highly Active Antiretroviral Therapy. Proc. Natl. Acad. Sci. USA.

[19] Pace M.J., Graf E.H., Agosto L.M., Mexas A.M., Male F., Brady T., Bushman F.D., O’Doherty U. (2012). Directly Infected Resting CD4+T Cells Can Produce HIV Gag without Spreading Infection in a Model of HIV Latency. PLoS Pathog..

[20] Sun N., Yau S.S.-T. (2022). In-Depth Investigation of the Point Mutation Pattern of HIV-1. Front. Cell. Infect. Microbiol..

[21] Motozono C., Mwimanzi P., Ueno T. (2010). Dynamic Interplay between Viral Adaptation and Immune Recognition during HIV-1 Infection. Protein Cell.

[22] Giombini E., Dolcetti R., Caccuri F., Selleri M., Rozera G., Abbate I., Bartolini B., Martorelli D., Faè D.A., Fiorentini S. (2014). Detection of HIV-1 Matrix Protein P17 Quasispecies Variants in Plasma of Chronic HIV-1-Infected Patients by Ultra-Deep Pyrosequencing. J. Acquir. Immune Defic. Syndr..

[23] Zani A., Messali S., Bugatti A., Uggeri M., Rondina A., Sclavi L., Caccuri F., Caruso A. (2024). Molecular Mechanisms behind the Generation of Pro-Oncogenic HIV-1 Matrix Protein P17 Variants. J. Gen. Virol..

[24] Giagulli C., D’Ursi P., He W., Zorzan S., Caccuri F., Varney K., Orro A., Marsico S., Otjacques B., Laudanna C. (2017). A Single Amino Acid Substitution Confers B-Cell Clonogenic Activity to the HIV-1 Matrix Protein P17. Sci. Rep..

[25] He W., Mazzuca P., Yuan W., Varney K., Bugatti A., Cagnotto A., Giagulli C., Rusnati M., Marsico S., Diomede L. (2019). Identification of Amino Acid Residues Critical for the B Cell Growth-Promoting Activity of HIV-1 Matrix Protein P17 Variants. Biochim. Biophys. Acta Gen. Subj..

[26] Giagulli C., Caccuri F., Zorzan S., Bugatti A., Zani A., Filippini F., Manocha E., D’Ursi P., Orro A., Dolcetti R. (2021). B-Cell Clonogenic Activity of HIV-1 P17 Variants Is Driven by PAR1-Mediated EGF Transactivation. Cancer Gene Ther..

[27] Liu X., Yu J., Song S., Yue X., Li Q. (2017). Protease-Activated Receptor-1 (PAR-1): A Promising Molecular Target for Cancer. Oncotarget.

[28] D’Ursi P., Rondina A., Zani A., Uggeri M., Messali S., Caruso A., Caccuri F. (2024). Molecular Mechanisms Involved in the B Cell Growth and Clonogenic Activity of HIV-1 Matrix Protein P17 Variants. Viruses.

[29] Dolcetti R., Giagulli C., He W., Selleri M., Caccuri F., Eyzaguirre L.M., Mazzuca P., Corbellini S., Campilongo F., Marsico S. (2015). Role of HIV-1 Matrix Protein P17 Variants in Lymphoma Pathogenesis. Proc. Natl. Acad. Sci. USA.

[30] Giagulli C., Magiera A.K., Bugatti A., Caccuri F., Marsico S., Rusnati M., Vermi W., Fiorentini S., Caruso A. (2012). HIV-1 Matrix Protein P17 Binds to the IL-8 Receptor CXCR1 and Shows IL-8-like Chemokine Activity on Monocytes through Rho/ROCK Activation. Blood.

[31] Caccuri F., Giordano F., Barone I., Mazzuca P., Giagulli C., Andò S., Caruso A., Marsico S. (2017). HIV-1 Matrix Protein P17 and Its Variants Promote Human Triple Negative Breast Cancer Cell Aggressiveness. Infect. Agent. Cancer.

[32] Caccuri F., Giagulli C., Bugatti A., Benetti A., Alessandri G., Ribatti D., Marsico S., Apostoli P., Slevin M.A., Rusnati M. (2012). HIV-1 Matrix Protein P17 Promotes Angiogenesis via Chemokine Receptors CXCR1 and CXCR2. Proc. Natl. Acad. Sci. USA.

[33] Caccuri F., Rueckert C., Giagulli C., Schulze K., Basta D., Zicari S., Marsico S., Cervi E., Fiorentini S., Slevin M. (2014). HIV-1 Matrix Protein P17 Promotes Lymphangiogenesis and Activates the Endothelin-1/Endothelin B Receptor Axis. Arterioscler. Thromb. Vasc. Biol..

[34] Basta D., Latinovic O., Lafferty M.K., Sun L., Bryant J., Lu W., Caccuri F., Caruso A., Gallo R., Garzino-Demo A. (2015). Angiogenic, Lymphangiogenic and Adipogenic Effects of HIV-1 Matrix Protein P17. Pathog. Dis..

[35] Folkman J. (2002). Role of Angiogenesis in Tumor Growth and Metastasis. Semin. Oncol..

[36] Al-Rawi M.A.A., Jiang W.G. (2011). Lymphangiogenesis and Cancer Metastasis. Front. Biosci..

[37] Caccuri F., Muraro E., Gloghini A., Turriziani O., Riminucci M., Giagulli C., Mastorci K., Fae’ D.A., Fiorentini S., Caruso A. (2019). Lymphomagenic Properties of a HIV P17 Variant Derived from a Splenic Marginal Zone Lymphoma Occurred in a HIV-Infected Patient. Hematol. Oncol..

[38] Carnero A., Blanco-Aparicio C., Renner O., Link W., Leal J.F.M. (2008). The PTEN/PI3K/AKT Signalling Pathway in Cancer, Therapeutic Implications. Curr. Cancer Drug Targets.

[39] Novoplansky O., Shnerb A.B., Marripati D., Jagadeeshan S., Abu Shareb R., Conde-López C., Zorea J., Prasad M., Ben Lulu T., Yegodayev K.M. (2023). Activation of the EGFR/PI3K/AKT Pathway Limits the Efficacy of Trametinib Treatment in Head and Neck Cancer. Mol. Oncol..

[40] Capobianchi M.R., Giombini E., Rozera G. (2013). Next-Generation Sequencing Technology in Clinical Virology. Clin. Microbiol. Infect..

[41] Perry C., Rayat A.C.M.E. (2021). Lentiviral Vector Bioprocessing. Viruses.

[42] Carroll V.A., Lafferty M.K., Marchionni L., Bryant J.L., Gallo R.C., Garzino-Demo A. (2016). Expression of HIV-1 Matrix Protein P17 and Association with B-Cell Lymphoma in HIV-1 Transgenic Mice. Proc. Natl. Acad. Sci. USA.

[43] Zentner I., Sierra L.-J., Fraser A.K., Maciunas L., Mankowski M.K., Vinnik A., Fedichev P., Ptak R.G., Martín-García J., Cocklin S. (2013). Identification of a Small-Molecule Inhibitor of HIV-1 Assembly That Targets the Phosphatidylinositol (4,5)-Bisphosphate Binding Site of the HIV-1 Matrix Protein. ChemMedChem.

[44] Iaria M.L., Fiorentini S., Focà E., Zicari S., Giagulli C., Caccuri F., Francisci D., Di Perri G., Castelli F., Baldelli F. (2014). Synthetic HIV-1 Matrix Protein P17-Based AT20-KLH Therapeutic Immunization in HIV-1-Infected Patients Receiving Antiretroviral Treatment: A Phase I Safety and Immunogenicity Study. Vaccine.

[45] Focà E., Iaria M.L., Caccuri F., Fiorentini S., Motta D., Giagulli C., Castelli F., Caruso A. (2015). Long-Lasting Humoral Immune Response Induced in HIV-1-Infected Patients by a Synthetic Peptide (AT20) Derived from the HIV-1 Matrix Protein P17 Functional Epitope. HIV Clin. Trials.

